# Coherent perfect absorption of nonlinear matter waves

**DOI:** 10.1126/sciadv.aat6539

**Published:** 2018-08-10

**Authors:** Andreas Müllers, Bodhaditya Santra, Christian Baals, Jian Jiang, Jens Benary, Ralf Labouvie, Dmitry A. Zezyulin, Vladimir V. Konotop, Herwig Ott

**Affiliations:** 1 Department of Physics and OPTIMAS Research Center, Technische Universität Kaiserslautern, Erwin Schrödinger Straße, 67663 Kaiserslautern, Germany.; 2 Graduate School Materials Science in Mainz, Staudinger Weg 9, 55128 Mainz, Germany.; 3 ITMO University, Saint Petersburg 197101, Russia.; 4 Deparatmento de Física and Centro de Física Teórica e Computacional, Faculdade de Ciências, Universidade de Lisboa, Campo Grande, Ed. C8, Lisboa 1749-016, Portugal.

## Abstract

Coherent perfect absorption is the complete extinction of incoming radiation by a complex potential in a physical system supporting wave propagation. The concept was proven for linear waves in a variety of systems including light interacting with absorbing scatterers, plasmonic metasurfaces, and graphene films, as well as sound waves. We extend the paradigm to coherent perfect absorption of nonlinear waves and experimentally demonstrate it for matter waves in an atomic Bose-Einstein condensate. Coherent absorption of nonlinear matter waves is achieved easier than its linear analogs because the strength of two-body interactions offers additional freedom for control. Implementation of the coherent perfect absorber of Bose-Einstein condensates paves the way toward broad exploitation of the phenomenon in nonlinear optics, exciton-polariton condensates, acoustics, and other areas of nonlinear physics. It also opens perspectives for designing atom lasers.

## Teaser

Eliminating atoms of a Bose-Einstein condensate in a lattice from one cell is coherent perfect absorption of the quantum liquid.

## INTRODUCTION

Coherent perfect absorption (CPA) is the complete extinction of incoming radiation by a complex potential embedded in a physical system supporting wave propagation. The phenomenon is based on destructive interference of transmitted and reflected waves. The concept was introduced (*1*) and observed experimentally (*2*) for light interacting with absorbing scatterers. CPA was also reported for plasmonic metasurfaces (*3*), graphene films (*4*), and sound waves (*5*). Technologically, CPA is used to design switching devices (*6*) and logic elements (*7*), in interferometry (*2*), and in many other applications (*8*). All these studies deal with perfect absorption of linear waves. Here, we extend the paradigm to a CPA of nonlinear waves and experimentally demonstrate it for matter waves with an atomic Bose-Einstein condensate (BEC). Conditions for CPA of matter waves can be satisfied easier than for its linear analogs because the strength of two-body interactions offers additional freedom for control. The observation of CPA of nonlinear matter waves paves the way toward a much broader exploitation of the phenomenon in nonlinear optics, exciton-polariton condensates, acoustics, and other areas of nonlinear physics.

**Fig. 1:**
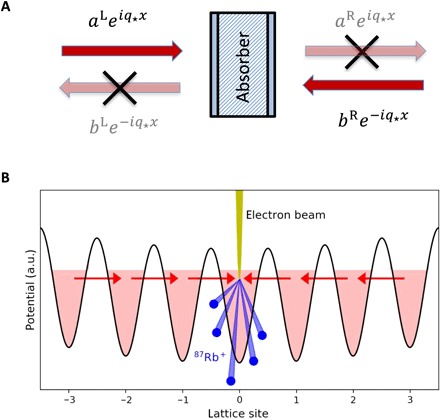
Working principle of CPA. (**A**) Incident radiation enters an absorber from two sides with a wave vector *q*_⋆_. Provided that the complex amplitudes *a*^L^ and *b*^R^ are chosen properly, no radiation is transmitted or reflected. (**B**) Experimental realization with a BEC in an optical lattice. The matter waves enter a lossy lattice site from both sides. The losses are realized with an electron beam, which removes the atoms. Because of the interactions between the atoms, the two incoming waves are nonlinear, while the absorption in the lossy site is linear. a.u., arbitrary units.

CPA is a delicate phenomenon requiring precise tuning of the absorber and of the relative phases of the incoming waves. When the respective conditions are met for a particular wave vector, the radiation incident from both sides is completely absorbed (Fig. 1A). CPA can be viewed as a time-reversed process to lasing (*1*, *2*), where the absorber is replaced by a gain medium and only outgoing radiation exists for a given wave vector. This time-reversed process is related to the mathematical notion of a spectral singularity (*9*), that is, to a wave vector at which the system can emit radiation with none incoming (*10*, *11*). Therefore, in the scattering formalism, the wavelength at which CPA occurs is called a time-reversed spectral singularity.

Recently, the concept of nonlinear CPA was introduced in optics (*12*–*15*). Such a device represents a nonlinear absorbing slab, sometimes with nontrivial composite internal structure (*16*, *17*), which is embedded in a linear medium and, thus, perfectly absorbs incident linear waves. Since, however, the propagating medium can itself be nonlinear, as is the case for an optical Kerr nonlinearity or an interacting BEC, the natural (and still open) question arises about the physical meaning of CPA in a nonlinear medium. In other words, what are the scattering properties of nonlinear waves interacting with a linear absorbing potential?

The implementation of CPA in a nonlinear medium offers several challenges that raise doubts about whether such a phenomenon can exist and whether it is physically meaningful. In such a setting, the “linear” arguments do not work: There is no well-defined transfer matrix connecting left and right incident waves [problems, where either the incident or the transmitted radiation is given, have different solutions (*18*)]; because of interactions among the modes, there exists no interference in the linear sense, and results on scattering of monochromatic waves do not give any more an answer on the scattering of wave packets used in experiments. Moreover, even if CPA can exist in a nonlinear medium, its realization is still questionable. There exists no general method of computing the system parameters, like the zeros of the transfer matrix elements in the linear case. Thus, tuning system parameters in situ might be the only possibility to realize CPA in nonlinear media. Furthermore, even the realization of plane waves may be practically impossible due to instabilities ubiquitous for nonlinear systems.

Here, we show that all the above challenges can be overcome: CPA for nonlinear waves does exist, can be observed experimentally, and can even be more easily achieved because of intrinsic nonlinearity. Theoretical indication for such CPA stems from the existence of stable constant amplitude currents in a nonlinear waveguiding circle with equal absorbing and lasing potentials (*19*). Experimental indication comes from recent experiments on driven dissipative Josephson systems (*20*).

## RESULTS

Here, we consider an atomic BEC residing in a periodic potential, realized by an optical lattice. The superfluid nature of the BEC allows for tunneling between the wells, while interatomic collisions lead to an intrinsic nonlinearity. One of the wells is rendered absorptive by applying an electron beam (*21*), which removes atoms from that well. The effective experimental system is sketched in Fig. 1B: A well with linear absorption, embedded between two tunneling barriers, is coupled at both ends to a nonlinear waveguide. For an introduction to the experimental techniques for manipulating ultracold atoms in optical potentials, the reader is referred to (*22*). Experimental details of the optical lattice, the preparation of the BEC, and the experimental sequence are given in Materials and Methods. The depth of the periodic potential and the number of atoms *N* in each lattice site (*N* ≈ 700) are chosen, such that we can apply the tight-binding approximation of mean field dynamics, when the condensate is described in terms of the density amplitudes ψ_*n*_(*t*) (*23*, *24*)

$$i\hbar \frac{d\psi_{n}}{dt}=-J(\psi_{n-1}+\psi_{n+1})+U{\left|\psi_{n}\right|}^{2}\psi_{n}-i\frac{\hbar \gamma }{2}\psi_{n}\delta_{n0}$$ (1)

Here, *n* enumerates the lattice sites, and γ ≥ 0 describes the dissipation strength applied to the site *n* = 0. Theoretically, such a system was first considered in (*25*) within the framework of the Gross-Pitaevskii equation. Later on, it was treated within the Bose-Hubbard model (*26*, *27*). Current states in BECs in the presence of dissipation and external drive were also studied theoretically in (*19*, *28*) and experimentally in (*20*, *29*, *30*).

Since CPA is a stationary process, we look for steady-state solutions of Eq. 1 in the form ψ_*n*_(*t*) = *e*^−*i*(μ/*ℏ*)*t*^*u*_*n*_, where all *u*_*n*_ are time-independent and μ is the chemical potential. First, we revisit the linear case corresponding to noninteracting atoms, $\tilde{\mu }{\tilde{u}}_{n}=-J({\tilde{u}}_{n-1}+{\tilde{u}}_{n+1})-i(\hbar \gamma /2){\tilde{u}}_{n}\delta_{n0}$, where we use tildes to emphasize the limit *U* = 0. In the absence of dissipation, that is, at γ = 0, the dispersion relation in the tight-binding approximation reads $\tilde{\mu }=-2J\text{cos}q$, where *q* ∈ [0, π] is the wave number. When dissipation is applied at *n* = 0, we consider the left ${\tilde{u}}_{n}^{L}=a^{L}e^{iqn}+b^{L}e^{-iqn}$ for *n* ≤ −1 and the right ${\tilde{u}}_{n}^{R}=a^{R}e^{iqn}+b^{R}e^{-iqn}$ for *n*≥ 1 solutions, where *a*^L^ and *b*^R^ (*a*^R^ and *b*^L^) are the incident (reflected) waves from left (L) and right (R), respectively (see Fig. 1). The transfer 2 × 2 matrix M with the elements *M*_*ij*_(*q*) is defined by the relation (*a*^R^, *b*^R^)^T^ = M (*a*^L^, *b*^L^)^T^, where T stands for transpose. Computing M (see Materials and Methods), one verifies that, for *q* = *q*_⋆_ and $q=q_{⋆}^{1}=\pi -q_{⋆}$ where

$$q_{⋆}=\;\text{arcsin}(\frac{\hbar \gamma }{4J})$$ (2)

the element *M*_11_ vanishes [*M*_11_(*q*_⋆_) = 0], while the other elements become *M*_21_(*q*_⋆_) = − *M*_12_(*q*_⋆_) = 1 and *M*_22_(*q*_⋆_) = 2. At these wave numbers, the problem admits a solution consisting of only incident waves, that is, *a*^L^ = *b*^R^ and *b*^L^ = *a*^R^ = 0. Thus, two CPA states occur for slow (*q* = *q*_⋆_) and fast ($q=q_{⋆}^{1}$) matter waves. The points *q*_⋆_ and $q_{⋆}^{1}$ are called time-reversed spectral singularities.

If instead of eliminating, one coherently injects atoms into the site *n* = 0, that is, γ < 0, Eq. 1 admits spectral singularities ${\bar{q}}_{⋆}=-q_{⋆}$ and ${\bar{q}}_{⋆}^{1}=-q_{⋆}^{1}$ at which $M_{22}({\bar{q}}_{⋆})=0$, while the other elements become $M_{12}({\bar{q}}_{⋆})=-M_{21}({\bar{q}}_{⋆})=1$ and $M_{11}({\bar{q}}_{⋆})=2$. Now, the solution *a*^R^ = *b*^L^ and *a*^L^ = *b*^R^ = 0 describes coherent wave propagation outside the “active” site, corresponding to a matter-wave laser. Since the change γ → − γ in Eq. 1 is achieved by applying the Wigner time reversal operator T: TΨ(r,t)=Ψ*(r,−t) where Ψ is an order parameter of the BEC, a coherent perfect absorber corresponds to a time-reversed laser (*1*).

The CPA solutions of Eq. 1 for linear waves have the following properties: They exist only for dissipation rates with γ ≤ γ_th_ = 4*J*/*ℏ*. The amplitude of the absorbed waves is constant in all sites, including the site where atoms are eliminated, and the group velocity at *q*_⋆_ is directly set by the decay rate: $v_{g}(q_{⋆})=d\tilde{\mu }/dq{|}_{q⋆}=2J\text{sin}q_{⋆}=\hbar \gamma /2$.

Bearing these properties in mind, we now turn to the nonlinear problem, setting *U* > 0 (repulsive interactions among the atoms). We search for a steady-state solution of Eq. 1 with a constant amplitude ρ in each lattice site. The requirement for the existence of only left and right incident waves can be formulated as $u_{n}^{L}=\rho e^{iqn}$ for *n* ≤ −1, $u_{n}^{R}=\rho e^{-iqn}$ for *n* ≥ 1, and *u*_0_ = ρ. This fixes μ = − 2*J* cos *q* + *Uρ*^2^, and the matching conditions at *n* = 0 imply that the steady-state solution exists only if *q* = *q*_⋆_ (and $q=q_{⋆}^{1}$) given by Eq. 2. Thus, we have obtained CPA for nonlinear matter waves, which still corresponds to the time-reversed laser. Indeed, as in the linear case, replacing the dissipation with gain (that is, inverting the sign of γ), one obtains the constant amplitude outgoing-wave solution: $u_{n}^{L}=\rho e^{-iq_{⋆}n}$ for *n* ≤ −1 and $u_{n}^{R}=\rho e^{iq_{⋆}n}$ for *n* ≥ 1.

One essential difference between linear and nonlinear CPA is particularly relevant for the experimental observation of the phenomenon: the stability of the incoming superfluid currents. The stability analysis (see Materials and Methods) shows that the nonlinearity qualitatively changes the result: Only slow currents (*q* = *q*_⋆_) can be perfectly absorbed, while the fast nonlinear currents ($q=q_{⋆}^{1}$) are dynamically unstable.

**Fig. 2:**
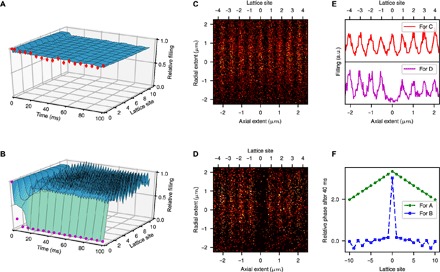
CPA in a BEC residing in an optical lattice. At *t* = 0, the elimination of atoms from the lattice site *n* = 0 starts. (**A** and **B**) Time evolution of the lattice filling in the CPA regime [γ ≈ 125 s^−1^ (A)] and above the breaking point [γ ≈ 1000 s^−1^ (B)]. Experimental data are shown as red points, and the numerical simulations are shown in blue. For clarity, we only show the first 10 sites on one side of the system. In (A), the density remains uniform across the lattice, which is the signature of CPA. In (B), the density at the dissipative site drops off rapidly, and no CPA can be observed. (**C** and **D**) Corresponding images of the atomic distribution in the lattice after the sequence. (**E**) Integrated atomic density of (C) and (D). (**F**) Unwrapped radian argument arg ψ_*n*_ at *t* = 40 ms for the solution shown in (A) (green curve) and (B) (blue curve). For all panels, we have *U*/*ℏ* = 2600 s^−1^, *J*/*ℏ* = 229 s^−1^. The error bars in (A) and (B) indicate the statistical error, resulting from the summation over 50 experimental runs.

The CPA solution is mathematically valid only in the infinite lattice because the absorption at the center must be compensated by steady particle fluxes incoming from infinity. However, the CPA phenomenon is structurally robust and can be observed as a quasi-stationary regime even in a finite lattice. To demonstrate this, we numerically simulated Eq. 1 with about 200 sites. The initial condition corresponds to the ground state of a BEC with an additional small harmonic confinement along the lattice direction. Figure 2 (A and B) shows the obtained behavior for dissipation strengths γ below (A) and above (B) the CPA breaking point γ_th_. Figure 2A shows that the solution rapidly enters a quasi-stationary regime where its density remains constant in space and is only weakly decaying in time due to an overall loss of atoms in the system. Above the breaking value (Fig. 2B), a strong decay sets in and the atomic density is not homogeneous in space any more. An important feature of CPA is the balanced superfluid currents toward the dissipative site, characterized by the distribution of phases, illustrated in Fig. 2F. The CPA regime manifests itself in the Λ-shaped phase profile whose slope is *q*_⋆_ for negative *n* and −*q*_⋆_ for positive *n*. This phase pattern is completely different when the system is not in the CPA regime: It is nearly constant, showing weak nonmonotonic behavior for positive and negative *n*, with a large jump at the central site.

Together with the numerical simulation, we also show in Fig. 2 the corresponding experimental results. The experimentally measured filling level of the dissipative site shows very good agreement with the numerical simulations: The atom number in the dissipated site is constant in time (Fig. 2A) and equal to all neighboring sites (Fig. 2C). This steady state is the experimental manifestation of CPA of matter waves. The CPA solution is established also for other values of the dissipation strength. This highlights the fact that the nonlinearity together with the dissipation generates an effective attractor dynamics toward the CPA solution. Increasing the dissipation above a critical value leads to a qualitative change in the behavior (Fig. 2, B and D). In accordance with the theoretical prediction, the occupation in the dissipated site rapidly drops and stays small. Hence, CPA can, indeed, only be observed in a finite parameter window.

**Fig. 3:**
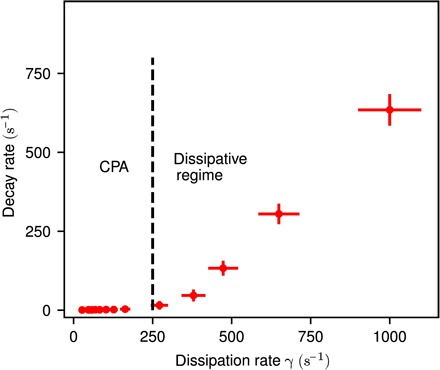
Experimentally measured decay rate of the filling level. Up to a critical dissipation strength γ_exp_ ≈ 250 s^−1^ (black dashed line), the filling level remains constant (compare Fig. 2A), corresponding to the CPA regime. Above this value, the dissipation dominates the dynamics, and the filling level decays exponentially (compare Fig. 2B). The statistical error of the decay rate is smaller than the size of the points; however, we estimate a 5% systematic error due to technical imperfections such as drifts of the electron beam current. The error in the dissipation rate originates from the calibration measurement (see Materials and Methods).

The theoretical results predict the transition from CPA and the non-CPA regime at γ_*th*_ = 4*J*/*ℏ*, above which no quasi–steady state is established anymore. In the experiment, a qualitatively similar situation occurs (Fig. 3). However the CPA regime breaks down at a lower dissipation rate of γ_exp_ ≈ *J*/*ℏ*. This can be explained by two factors. First, the transverse extension of each lattice site, not fully accounted for by the tight-binding approximation (Eq. 1), makes the condensate vulnerable against transverse instabilities (*31*), which can develop at smaller γ than predicted by Eq. 1. The second factor relates to the way the experiment is conducted. At *t* = 0, the condensate is loaded in a lattice and is characterized by the chemical potential, which is determined by the trap geometry and by the filling of each lattice site. When the elimination of atoms starts, the system is induced in an unstable regime. Quasi-stationary behavior is only possible if the chemical potential is not changed appreciably under the action of the dissipation. This requires the filling of the central site due to tunneling from the neighboring ones to be fast enough to compensate the loss of atoms. Thus, the tunneling time estimated as τ_tun_ ~*ℏ*/*J* should be of the order of or smaller than the inverse loss rate γ^−1^; otherwise, the induced thermodynamical equilibrium at *t* = 0 cannot be compensated by the incoming superfluid currents, and the collective dynamics described by Eq. 1 cannot be established. This gives an estimate γ ~ *J*/*ℏ* for the threshold dissipation rate.

## CONCLUSION

Our results present the proof of concept of the CPA paradigm for nonlinear waves. The experimental setting explored here can be straightforwardly generalized to BECs of other types such as spin-orbit–coupled, fermionic, and quasi-particle ones, and furthermore to other branches of physics, including nonlinear optics of Kerr media and acoustics. Our system can also be exploited as a platform for studying superfluid flows in a linear geometry (which is an alternative to most commonly used annular traps), as well as for understanding the fundamental role of Bogoliubov phonons in stabilizing quantum states. Since CPA can be viewed as time-reversed lasing, the reported experimental results pave the way to implementing a laser for matter waves, for which elimination of atoms from the central site should be replaced by injecting atoms. Furthermore, the observation of CPA in nonlinear media, and possible lasing of matter waves, can be viewed as an additional element for the rapidly developing area of quantum technologies based on atomtronics (*32*). The reported results also open the possibility of using CPA regimes in nonlinear optical circuits.

In the general context of scattering by dissipative potentials (*33*), given the fact that the atomic interactions can be tuned by a Feshbach resonance, a linear spectral singularity can be experimentally realized by starting from the nonlinear case and subsequently reducing the interactions to zero adiabatically. Such a scenario explicitly exploits the attractor nature of the CPA solution. Being an attractor in an essentially nonlinear system, CPA can serve as a mechanism to control superfluid flow parameters, such as the chemical potential, superfluid velocity, or sound velocity, in a particularly simple way.

## MATERIALS AND METHODS

### The transfer matrix

To compute the transfer matrix M, we denoted the solution in the point *n* = 0 by *u*_0_ and considered the equation

$$\tilde{\mu }{\tilde{u}}_{n}=-J({\tilde{u}}_{n-1}+{\tilde{u}}_{n+1})-i\frac{\hbar \gamma }{2}{\tilde{u}}_{n}\delta_{n0}$$ (3)

describing stationary currents, in the points *n* = 0 and *n* = ±1 using the explicit forms for the waves in the left ${\tilde{u}}_{n}^{L}=a^{L}e^{iqn}+b^{L}e^{-iqn}$ (*n* ≤ −1) and the right ${\tilde{u}}_{n}^{R}=a^{R}e^{iqn}+b^{R}e^{-iqn}$ (*n* ≥ 1) half-space. From the equation, with *n* = 0 and using the expression for the chemical potential $\tilde{\mu }=-2J\text{cos}q$, we obtained *u*_0_

$$u_{0}=\frac{2J}{4J\text{cos}q-i\hbar \gamma }(a^{L}e^{-iq}+b^{L}e^{iq}+a^{R}e^{iq}+b^{R}e^{-iq})$$ (4)

With this expression, the equations at *n* = ±1 are transformed to a linear algebraic system, which is solved for the pair (*a*^R^, *b*^R^), giving their expressions through (*a*^L^, *b*^L^), thus determining the transfer matrix

$$M_{11}=\frac{4J\text{sin}q-\hbar \gamma }{4J\text{sin}q},M_{12}=-\frac{\hbar \gamma }{4J\text{sin}q},M_{21}=\frac{\hbar \gamma }{4J\text{sin}q},M_{22}=\frac{4J\text{sin}q+\hbar \gamma }{4J\text{sin}q}$$ (5)

### Stability analysis

We analyzed the stability for a BEC within the framework of the discrete model

$$i\hbar \frac{d\psi_{n}}{dt}=-J(\psi_{n-1}+\psi_{n+1})+U{\left|\psi_{n}\right|}^{2}\psi_{n}-i\frac{\hbar \gamma }{2}\psi_{n}\delta_{n0}$$ (6)

and required that the left and right incident superfluid currents have to be stable. Their stability is determined by the stability of the corresponding Bogoliubov phonons on an infinite homogeneous lattice (that is, without applied removal of atoms). The stability of the homogeneous lattice is found using the substitution

$$\psi_{n}(t)=e^{-i(\mu /\hbar )t+iqn}(\rho +v_{n}e^{-i\omega t+ikn}+w_{n}^{*}e^{i\mathrm{\omega *}*t-ikn})$$ (7)

where ρ > 0 characterizes the uniform density, and |*v*_*n*_| , |*w*_*n*_| ≪ ρ are small perturbations. Linearizing Eq. 6 (with γ = 0) with respect to *v*_*n*_ and *w*_*n*_, we found two dispersion branches

$$\hbar \omega_{\pm }(q,k)=2J\;\text{sin}(k)\text{sin}(q)\pm 2\text{sin}(\frac{k}{2})\sqrt{2J\text{cos}(q)[U\rho^{2}+2J\text{cos}(q){\text{sin}}^{2}(\frac{k}{2})]}$$ (8)

Consider now a positive scattering length, *U* > 0, which corresponds to the experiments reported here. One can then identify the stability domain for Bogoliubov phonons and, hence, the stability of the superfluid current, requiring ω_±_ to be real for the given *q* and all real *k*. This results in the constraint 0 ≤ *q* < π/2, that is, only slow currents are dynamically stable.

### Experimental setup

We used a BEC of ^87^Rb with about 45 × 10^3^ atoms in a single-beam dipole trap realized by a CO_2_ laser (maximum power, 10 W; beam waist, 30 μm). The condensate is cigar-shaped and has dimensions of 80 μm × 6 μm × 6 μm. We then loaded the BEC into a one-dimensional optical lattice created by two blue detuned laser beams (λ = 774 nm; beam waist, 500 μm) crossed at an angle of 90°. The linear polarization of both laser beams was along the same direction, such that the interference pattern was maximally modulated. The resulting lattice has a period of *d* = 547 nm. The trap frequencies in a lattice site are *ν*_*r*_ = 165 Hz (transverse direction) and *ν*_*z*_ = 12 kHz (lattice direction). Each site contains a small, pancake-shaped BEC with about 700 atoms (value in the center of the trap). The total number of lattice sites is about 200. The lattice depth *V*_0_ in units of the recoil energy *E*_*r*_ = π^2^*ℏ*^2^/(2*md*^2^) (*m* is the mass of the atom) is given by *V*_0_ = 10*E*_*r*_. An electron column, which is implemented in our experimental chamber, provides a focused electron beam, which is used to introduce a well-defined local particle loss as a dissipative process in one site of the lattice. To ensure a homogeneous loss process over the whole extension of the lattice site, we rapidly scanned the electron beam in the transverse direction (3-kHz scan frequency) with a sawtooth pattern. To adjust the dissipation strength γ, we varied the amplitude of the scan pattern. An image of the experimental chamber together with a sketch of the optical trapping configuration is provided in fig. S1.

### Why the lattice is necessary

As mentioned in the main text, the superfluid currents under localized dissipation were studied previously in inhomogeneous BECs (*22*, *25*), where no CPA was observed. Mathematical solutions of the Gross-Pitaevskii equation with localized dissipation describing such models can, however, be found. Such solutions are stable and have stationary amplitudes. Consider the stationary Gross-Pitaevskii equation with strongly localized dissipation modeled by the Dirac delta function γ_0_δ(*x*), where γ_0_ is a positive constant, without any optical lattice

$$\mu \psi =-\psi_{xx}-i\gamma_{0}\delta (x)\psi +g{\left|\psi \right|}^{2}\psi$$ (9)

Here, *g* > 0. One can verify that the function

$$\psi =\rho_{0}e^{-i\gamma_{0}x\text{sign}(x)/2}$$ (10)

is a solution of Eq. 9 with the chemical potential $\mu =g\rho_{0}^{2}+\gamma_{0}^{2}/4$.

This raises questions about the role of the optical lattice and about its necessity for realizing CPA experimentally. The answer resides in the way of exciting the CPA regime. A strictly homogeneous background density can only exist if the dissipation is point-like (described by the Dirac delta function δ) and, thus, experimentally unrealistic. Any finite-size, even very narrow, dissipation generates Bogoliubov phonons at the instant it is applied. In the continuous model, the phonons can propagate with arbitrary group velocity, contrary to the lattice described in the tight-binding model. Switching on dissipation therefore induces an extended domain of the condensate in a dynamical regime, and fast matter waves propagating outward the dissipation domain cannot be stabilized by the incoming flows. Thus, the lattice, on the one hand, creates conditions where the dissipation is effectively point-like (that is, applied to a single cell) and, on the other hand, limits the group velocity of the phonons, allowing the establishment of the equilibrium state.

### Details of numerical simulations

In the numerical simulations, we used the following model that corresponds to the Gross-Pitaevskii equation from the main text with an additional weak parabolic confinement α*n*^2^ψ_*n*_, which models the optical dipole trap potential used in the experimental setup

$$i\hbar \frac{d\psi_{n}}{dt}=-J(\psi_{n-1}+\psi_{n+1})+U{\left|\psi_{n}\right|}^{2}\psi_{n}-\frac{i\gamma \hbar }{2}\delta_{n0}\psi_{n}+\alpha n^{2}\psi_{n}$$ (11)

The coefficient α determines the strength of the parabolic trapping and amounts to

$$\alpha =\frac{m\omega^{2}d^{2}}{2\hbar }=0.98s^{-1}$$ (12)

where *m* = 1.44 ⋅ 10^−25^ g is the mass of the atom, *d* = 547 nm is the lattice period, and ω = 2π ⋅ 11 Hz is the axial trapping frequency of the dipole trap. As in the main text, for other parameters, we have *J*/*ℏ* = 229 s^−1^ and *U*/*ℏ* = 2600 s^−1^.

To convert Eq. 11 in the form suitable for numerical calculations, we divided each term by *J* and introduced the “new time” τ = (*J*/*ℏ*)*t* that transforms Eq. 11 to

$$i\frac{d\psi_{n}}{d\tau }=-(\psi_{n-1}+\psi_{n+1})+\tilde{U}{\left|\psi_{n}\right|}^{2}\psi_{n}-\frac{i\tilde{\gamma }}{2}\delta_{n0}\psi_{n}+\tilde{\alpha }n^{2}\psi_{n}$$ (13)

where *Ũ* = *U*/*J* ≈ 11, $\tilde{\alpha }=\alpha /J\approx 0.004$, and $\tilde{\gamma }=\gamma \hbar /J$.

For $\tilde{\gamma }=0$, Eq. 13 has an approximate stationary ground-state Thomas-Fermi solution

$$\psi_{n}=e^{-i(\tilde{U}\rho^{2}-2)t}w_{n},\;w_{n}=\{\begin{array}{cc} \sqrt{\rho^{2}-\tilde{\alpha }n^{2}/\tilde{U}}, & |n|<N_{\text{TF}}\\ 0, & |n|>N_{\text{TF}} \end{array}$$ (14)

where the “discrete Thomas-Fermi radius” *N*_TF_ is determined by the condition $\tilde{U}\rho^{2}-\tilde{\alpha }N_{\text{TF}}^{2}=0$, and ρ^2^ = 1 is the normalized background density, that is, |*w*_*n*_|^2^ ≈ 1 in the central region. In our case, *N*_TF_ ≈ 50. We solved Eq. 13 for ψ_*n*_(τ) on the grid of 201 sites *n* = −100,…, 100, where *n* = 0 corresponds to the site with the losses and is subject to the zero boundary conditions ψ_−100_(τ) = ψ_100_(τ) = 0.

For the initial condition, we used the ground-state Thomas-Fermi distribution from Eq. 14: ψ_*n*_(*t* = 0) = *w*_*n*_. As discussed in the main text, in the parametric range corresponding to the existence of the CPA, the initial condition rapidly evolves to the quasi-stationary CPA solution characterized by the almost uniform density in the central region (*n* = −10, …, 10), whereas, in the absence of the CPA regime, the initial condition rapidly develops strong instability in the central region.

## Supplementary Material

http://advances.sciencemag.org/cgi/content/full/4/8/eaat6539/DC1

## SUPPLEMENTARY MATERIALS

Supplementary material for this article is available at http://advances.sciencemag.org/cgi/content/full/4/8/eaat6539/DC1 Fig. S1. Photograph of the vacuum chamber and sketch of the optical trapping scheme.
