# Supplementary material for: Coherent perfect absorption of nonlinear matter waves
Source: Sci Adv. 2018 Aug 10;4(8):eaat6539. doi: 10.1126/sciadv.aat6539 (PMC6086614; doi:10.1126/sciadv.aat6539)
Supplement: http://advances.sciencemag.org/cgi/content/full/4/8/eaat6539/DC1 [file supp_4_8_eaat6539__index.html]

Science Advances | Science Advances

## Supplementary Materials

**This PDF file includes:**

- Fig. S1. Photograph of the vacuum chamber and sketch of the optical trapping scheme.

Download PDF

**Files in this Data Supplement:**

- Adobe PDF - aat6539\_SM.pdf
