# Supplementary material for: Coherent perfect absorption of nonlinear matter waves
Source: Sci Adv. 2018 Aug 10;4(8):eaat6539. doi: 10.1126/sciadv.aat6539 (PMC6086614; doi:10.1126/sciadv.aat6539)
Supplement: http://advances.sciencemag.org/cgi/content/full/4/8/eaat6539/DC1 [file aat6539_SM.pdf]

Science Advances
AAAS

advances.sciencemag.org/cgi/content/full/4/8/eaat6539/DC1

Supplementary Materials for

Coherent perfect absorption of nonlinear matter waves

Andreas Müllers, Bodhaditya Santra, Christian Baals, Jian Jiang, Jens Benary, Ralf Labouvie, Dmitry A. Zezyulin, Vladimir V. Konotop*, Herwig Ott*

*Corresponding author. Email: vvkonotop@fc.ul.pt (V.V.K.); ott@physik.uni-kl.de (H.O.)

Published 10 August 2018, *Sci. Adv.* **4**, eaat6539 (2018)
DOI: 10.1126/sciadv.aat6539

This PDF file includes:

Fig. S1. Photograph of the vacuum chamber and sketch of the optical trapping scheme.

This PDF file includes:

Figure S1

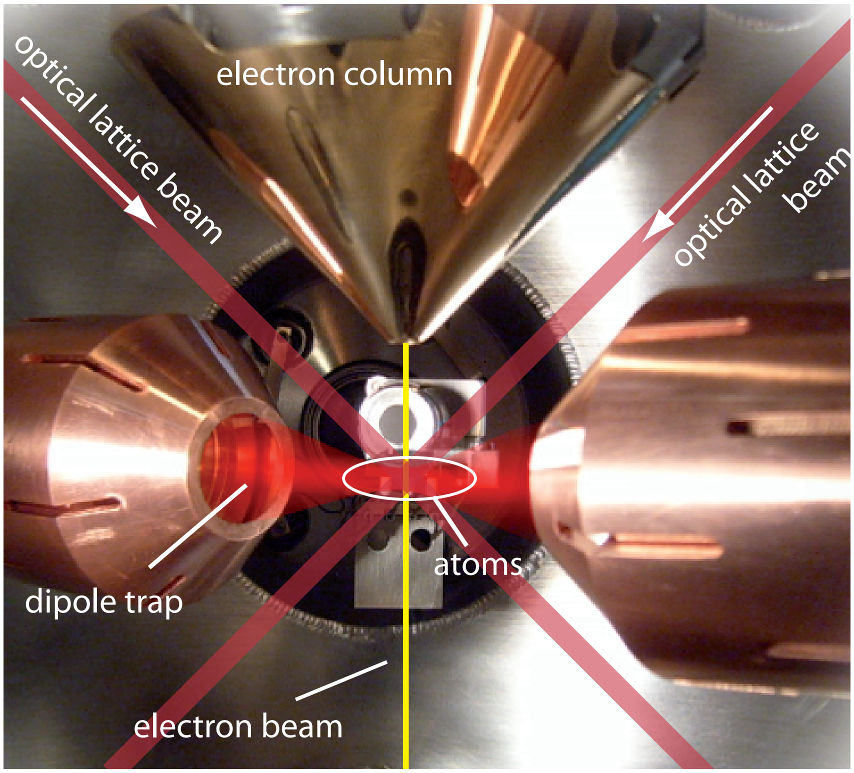

Fig. S1. Photograph of the vacuum chamber and sketch of the optical trapping scheme. The Bose-Einstein condensate is prepared in a single beam optical dipole trap. The periodic potential is created by two interfering optical lattice beams, which create a standing light wave at the position of the atoms. The combination of both potentials results in the physical situation as depicted in Fig. 1B. An electron column provides a focused electron beam, which is pointed at one of the potential wells. Scattering processes between the electrons and the ultracold atoms lead to local loss (absorption) of the atoms. The size of image section is about 15 cm $\times$ 15 cm.
